# Effect of seasonal influenza vaccination on influenza symptom severity among children in Hutterite communities: Follow‐up study of a randomized trial

**DOI:** 10.1111/irv.12689

**Published:** 2019-11-08

**Authors:** Shannon E. Brent, Eleanor Pullenayegum, Margaret L. Russell, Mark Loeb

**Affiliations:** ^1^ Michael G. DeGroote School of Medicine McMaster University Hamilton ON Canada; ^2^ The Hospital for Sick Children University of Toronto Toronto ON Canada; ^3^ Department of Community Health Sciences Cumming School of Medicine The University of Calgary Calgary AB Canada; ^4^ Departments of Pathology and Molecular Medicine and Health Research Evidence and Impact Institute for Infectious Disease Research McMaster University Hamilton ON Canada

**Keywords:** influenza, randomized trial, vaccination

## Abstract

**Background:**

We investigated whether influenza vaccination reduces symptom severity among children who develop laboratory‐confirmed influenza, and whether this association differed between influenza vaccine formulations.

**Methods:**

We performed a retrospective cohort study using data from two blinded cluster randomized control trials of influenza vaccines in Hutterite colonies. In trial 1, children received trivalent inactivated influenza vaccine (TIV) or hepatitis A vaccine. In trial 2, children received trivalent live attenuated (TLAIV) or TIV. We assessed four outcomes (total number of symptoms, number of respiratory symptoms, number of systemic symptoms, and duration of symptoms) among children with PCR‐confirmed influenza. We utilized two‐sample t tests to quantify the relationship between vaccine group and outcome. We performed multivariable strain‐specific analyses, controlling for age and season.

**Results:**

*TIV vs. Hep A vaccine:* Among vaccinated children, 200 confirmed influenza infections were observed across 3014 person‐seasons. Vaccine type (TIV vs. Hep A vaccine) did not significantly affect the number of respiratory or systemic symptoms, nor duration of symptoms (*P* > .05). *TLAIV vs. TIV:* Among 1186 children who received a study vaccine, 166 confirmed influenza infections were observed. TLAIV recipients experienced fewer total, respiratory, and systemic symptoms compared to TIV recipients (*P* < .05 for all). TLAIV‐associated attenuation of symptom severity was observed in influenza B or A/H1N1 infections, but not H3.

**Conclusions:**

Seasonal influenza vaccine did not consistently attenuate symptom severity in the context of vaccine failure; however, TLAIV offered superior severity attenuation compared to TIV. Our results challenge the dictum that influenza vaccine reduces the severity of symptoms even when the vaccine fails to prevent influenza.

## 1 INTRODUCTION

Seasonal influenza is a viral, acute respiratory illness that causes an estimated three to five million symptomatic cases and 290 000 to 645 000 deaths per year, globally.^1^ Influenza usually manifests as a sudden onset of fever, cough, headache, malaise, rhinorrhea, and pharyngitis.^2^ Illness lasts approximately one week and is typically self‐limiting without the need for medical attention. Despite this, high‐risk populations, such as young children, elderly individuals, people with chronic co‐morbidities, and immunocompromised individuals, can experience severe influenza‐related morbidity and mortality.^3^ Annual vaccination confers protection against seasonal influenza infection.^4^ When an individual is vaccinated against seasonal influenza, but develops a seasonal influenza infection, this is defined as a vaccine failure.^5^, ^6^ It follows that vaccine failures occur at a higher rate when vaccine effectiveness is low, or when the host cannot launch a protective antibody response.^6^, ^7^ Influenza vaccine effectiveness is variable and is often dependent on host (eg, age, health status) and epidemiological factors (eg, vaccine coverage, match to circulating strains).^8^ The type of influenza vaccine administered may also affect vaccine efficacy. Live attenuated influenza vaccines (TLAIV) were shown in early studies to be more efficacious than inactivated influenza vaccines (TIV) in children^9^, ^10^; however, more recent studies have not supported this finding.^11^

The US Centers for Disease Control and Prevention states that those vaccinated who develop a seasonal influenza infection may experience a milder course of disease as a result of vaccination.^12^ This statement is supported by evidence from studies that show reductions in upper respiratory symptoms, overall symptom severity,^13^, ^14^ and hospitalization rates in vaccinated individuals where vaccine failure occurred.^15^ Current evidence on vaccine‐attenuated symptom severity in patients who present with influenza‐like illness (ILI) has three main limitations. First, studies operationalize illness severity in different ways. Second, most studies have utilized test‐negative design. In brief, test‐negative studies typically identify a study population based on symptomatology and compare the characteristics (eg, severity of symptoms) of those who have a positive seasonal influenza test with those who do not;^16^ however, there are limitations to test‐negative studies. These studies can be vulnerable to substantial selection bias and confounding bias when estimating vaccine effectiveness.^17^ Third, studies to date have focused largely on adult in‐patient populations.^14^, ^15^ The relationship between seasonal influenza vaccination and symptom severity in the context of vaccine failure has been assessed using prospective data for children with influenza B infections,^6^ but to date has not been validated more broadly across other influenza strains, or across influenza vaccine formulations.

Utilizing data from two cluster randomized trials of influenza vaccine in Canadian Hutterite communities, we sought to understand whether seasonal influenza vaccination attenuates the severity of symptoms in the context of vaccine failure. Furthermore, we investigated whether this association differed between those who received the live attenuated influenza virus vaccine (TLAIV) and those who received the inactivated influenza vaccine (TIV).

## 2 METHODS

### 2.1 Study design and data collection

We performed a retrospective cohort study using data from two blinded cluster randomized control trials conducted across Hutterite colonies in Alberta, Saskatchewan, and Manitoba between 2008 and 2015. Both studies assessed the impact of immunizing children against seasonal influenza on community‐level infection rates.

Study 1 was a three‐year cluster randomized control trial where children within colonies received either TIV or hepatitis A vaccine as control. Throughout the three‐year trial, children aged 36 months to 15 years who received a study vaccine (hepatitis A or TIV), as well as other community members who did not receive study vaccine, were followed prospectively. Children randomized to a vaccination group were followed for a total of 3014 person‐seasons (1554 person‐seasons for TIV, 1460 person‐seasons for hepatitis A vaccine), and non‐vaccine recipients were followed for a total of 7971 person‐seasons across 65 colonies in Alberta, Manitoba, and Saskatchewan. Individuals with high‐risk conditions (eg, immunocompromise, pregnancy, diabetes) were also vaccinated with TIV at baseline regardless of age and followed prospectively.

Study 2 was a three‐year cluster randomized TIV‐controlled study, where children within colonies received either TLAIV or TIV. All participants were blinded to their colony's vaccine allocation. As above, the study followed children aged 36 months to 15 years who received study vaccine, community members who did not receive a study vaccine, and individuals with high‐risk conditions that were vaccinated at baseline. Over three seasons, 1186 children who received a study vaccine and 3425 community members who did not receive study vaccine across 52 Hutterite colonies in Alberta and Saskatchewan were followed forward. The complete methodology and results of the Hutterite community randomized trials have been published previously.^11^, ^18^

In both study 1 and study 2, the beginning of the follow‐up period for each influenza season was defined by the identification of >1 polymerase chain reaction (PCR)‐confirmed cases in two consecutive weeks in a sentinel site. Follow‐up for each influenza season concluded in a given health region after two consecutive weeks with zero laboratory‐confirmed influenza cases reported.

Throughout the follow‐up period, research nurses assessed vaccinated and non‐vaccinated participants twice a week. Using a standardized survey, nurses collected data on self‐reported signs and symptoms of ILI, including fever (≥38.0°C), cough, nasal congestion, sore throat, headache, sinus problems, muscle aches, fatigue, ear ache, or chills. The exact date of symptom onset was recorded. Nasopharyngeal specimens were obtained from any participant reporting 2 or more symptoms and were sent for real‐time reverse transcriptase polymerase chain reaction for viral genotyping.

### 2.2 Inclusion criteria

Study 1 was conducted over three influenza seasons between 2008 and 2011. Of the study participants followed prospectively, our analyses included children aged 36 months to 15 years of age from the original randomized control trials who (a) had a PCR‐confirmed seasonal influenza or pandemic (pH1N1) influenza infection during the study period and (b) received either the study influenza vaccine or a hepatitis A vaccine (control). We used November 1 as an age cut‐off at 15 years. We included all PCR‐confirmed influenza infections that were observed within the study period. If more than one confirmed infection occurred in the same participant over the three influenza seasons studied, each infection was considered an independent observation in the cohort. We assessed whether our results were robust to a violation of this assumption by performing a sensitivity analysis, using only the first infection for each participant. Subsequent infections occurring in any season were excluded from this sensitivity analysis. Given that our population of interest included only those who developed a PCR‐confirmed influenza infection in the context of vaccine failure, participants who were not vaccinated and developed an influenza infection were also excluded.

Study 2 was conducted over three influenza seasons between 2012 and 2015. As above, our analyses were conducted on children and adolescents aged 36 months to 15 years of age from the original randomized control trial who (a) had a PCR‐confirmed influenza infection during the study period, and (b) had been randomized to receive trivalent live attenuated influenza vaccine (TLAIV) or the trivalent inactivated influenza vaccine (TIV) and where the age cut‐off used was November 1 at 15 years. We included all PCR‐confirmed seasonal influenza infections observed throughout the study period for children who received either TLAIV or TIV within the study.

### 2.3 Outcomes

We identified two main outcomes to quantify the course of disease: number of symptoms and duration of symptoms. Given the potential lag between symptom onset and laboratory confirmation of influenza infection, we used symptom data collected by study nurses 7 days prior to the nasopharyngeal PCR sample collection date and assessed symptom data up to 14 days following the positive PCR result.

We counted the total number of individual symptoms reported during the 21‐day window. We then divided symptoms into two categories: respiratory (consistent with either upper and/or lower respiratory tract infection) or systemic. Total symptoms, respiratory symptoms, and systemic symptoms have been shown to correlate with cytokine titers in nasal lavage samples.^19^ In our analyses, respiratory symptoms included cough, sore throat, runny nose, sinus issues, and ear infection. Systemic symptoms included fever, headache, muscle aches, and fatigue. Symptomatic days where the participant reported at least one symptom did not have to be consecutive over the 21‐day follow‐up period and were summed to calculate duration of symptoms.

### 2.4 Statistical analyses

For both study 1 and study 2, we compared demographic characteristics of children and adolescents aged 36 months to 15 years randomized to each vaccine group using Pearson chi‐square testing for categorical variables, and Welch two‐sample t tests for continuous variables. For both studies, we stratified each cohort by vaccine group and used Welch two‐sample *t* tests, assuming unequal variances between groups, to assess the association between vaccine allocation and infection severity outcomes. Given the presence of repeat infections within individuals, we assessed the robustness of our results to the assumption that all infections are independent by performing a sensitivity analysis, using only the first infection for each participant.

To assess the influence of influenza stain type on the relationship between vaccination and infection severity, we performed multivariable strain‐specific analyses for influenza B, H3, H1N1, and pH1N1, controlling for age. We included a categorical interaction term for influenza season to assess whether the relationship between influenza strain type and each infection severity outcome changed based on the antigenic match of the influenza vaccine to the circulating strains each year.

## 3 RESULTS

### 3.1 Study 1: TIV vs Hepatitis a vaccine

Over three influenza seasons between October 2009 and May 2012, participating individuals were monitored for influenza symptoms across 39 Hutterite colonies in Alberta, Manitoba, and Saskatchewan. Characteristics of the original study population have been outlined previously.^18^, ^20^ Our primary analyses included children from 36 months to ≤15 years of age who were randomized to a vaccine group. Over three seasons, 200 PCR‐confirmed influenza infections occurred among 190 vaccinated (TIV or hepatitis A) individuals across 28 Hutterite colonies.

The demographic characteristics of children randomized to a vaccine group (TIV or hepatitis A) with PCR‐confirmed influenza infections are shown in Table 1. There were 122 infections in season 1, 21 infections in season 2, and 57 infections in season 3. All infections in season 2 were pandemic H1N1 infections. The average age at time of infection was 9.04 years (minimum = 3.10 years, maximum = 15.0 years, SD = 3.31 years), and 54.5% were female. The demographic characteristics of randomized children with a PCR‐confirmed influenza infection were similar in the two vaccine groups (Table 1); however, there were more males in the hepatitis A group than in the TIV group (*P* = .011), and the type of influenza strain causing the infection (pH1N1, H1, H3, or B) was significantly associated with vaccine group (*P* < .001).

**Table 1:** Demographic characteristics of PCR‐confirmed influenza infections among children from 2008 to 2011, across 28 Hutterite colonies

	All	Trivalent Inactivated Influenza Vaccine (TIV)	Hepatitis A Vaccine	*P*
	N = 200	N = 75	N = 125	
Age, mean (SD), y	9.04 (3.31)	8.86 (3.06)	9.15 (3.46)	.55
Male sex, N (%)	91 (45.5)	25 (33.3)	66 (52.8)	.011
Season (%)				
2008‐2009	122 (61.0)	46 (61.3)	76 (60.8)	.242
2009‐2010	21 (10.5)	11 (14.7)	10 ( 8.0)	
2010‐2011	57 (28.5)	18 (24.0)	39 (31.2)	
PCR‐confirmed Flu Strain (%)				
B	95 (47.5)	45 (60.0)	50 (40.0)	<.001
pH1N1	30 (15.0)	20 (26.7)	10 ( 8.0)	
Seasonal H1	17 (8.5)	0 ( 0.0)	17 (13.6)	
Seasonal H3	55 (27.5)	9 (12.0)	46 (36.8)	
Unknown A	3 (1.5)	1 ( 1.3)	2 ( 1.6)	

Among children with a PCR‐confirmed influenza infection, 125 received hepatitis A vaccination, and 75 received trivalent inactivated influenza vaccination prior to the commencement of influenza season. The average duration, total number of symptoms, number of respiratory symptoms, and number of systemic symptoms for the TIV group versus the hepatitis A vaccine group are presented in Table 2. We did not observe a significant effect of vaccine group on duration of symptoms (*P* = .144, mean difference = 0.34 days, 95% CI: −0.12 to 0.81 days), or overall number of symptoms (*P* = .984, mean difference = 0 symptoms, 95% CI: −0.53 to 0.52 symptoms). Similarly, when symptoms were categorized by type, vaccine group did not have a significant effect on the number of respiratory symptoms (*P* = .539, mean difference = 0.09 respiratory symptoms, 95% CI: −0.38 to 0.20 respiratory symptoms) or the number of systemic symptoms (*P* = .663, mean difference = 0.086 systemic symptoms, 95% CI: −0.30 to 0.47 symptoms). Our sensitivity analysis demonstrated that excluding repeat infections within individuals did not change the results of our analysis (results not shown); therefore, all infections among children randomized to a vaccine group across three seasons were treated as independent and retained in the analysis.

**Table 2:** Symptom severity and duration, stratified by vaccine group, for PCR‐confirmed influenza infections among children from 2008 to 2011

	Intervention Group		95% CI	*P*
	Trivalent Inactivated Influenza Vaccine (TIV)	Hepatitis A Vaccine		
Number of symptoms, mean (SD)	3.93	3.93	−0.534 to 0.523	.984
Number of respiratory symptoms, mean (SD)	2.35	2.26	−0.381 to 0.200	.539
Number of systemic symptoms, mean (SD)	1.59	1.67	−0.301 to 0.472	.663
Duration, mean (SD)	2.68	3.02	−0.119 to 0.807	.144

Our strain‐specific multivariable model analysis demonstrated that when controlling for age, the relationship between vaccine group and duration of illness among children with influenza B varied significantly across seasons (*P* = .036). In Season 1, vaccine group was not significantly associated with duration for influenza B infections; however, in Season 3, children who received an influenza vaccine experienced a significantly shorter duration of illness with influenza B, compared to those who received the hepatitis A vaccine (data not shown). Vaccine group was not significantly associated with the duration or number symptoms (respiratory, systemic, or total) in those with influenza H3 (*P* > .05 for all outcomes); this relationship did not vary significantly by season (*P* = .059). Children vaccinated with TIV who developed a pH1N1 infection experienced a statistically significant greater number of total symptoms (*P* = .022), systemic symptoms (*P* = .023), and respiratory symptoms (*P* = .0233) than those who received the hepatitis A vaccine (Table 3). For seasonal H1N1, all infections occurred in Season 1 among those who were randomized to the hepatitis A vaccine group; therefore, we were unable to assess the effect of vaccine group and season on our outcomes of interest.

**Table 3:** Multivariable linear regression model output for all PCR‐confirmed influenza pH1N1 infections among children (N = 30) from 2008 to 2011, adjusted for age

Predictors	Estimates	95% CI	*P*
Number of symptoms			
(Intercept)	0.33	−2.01 to 2.68	.782
TIV^a^	1.35	0.26 to 2.43	.022
Age	0.21	0.01 to 0.40	.053
Number of respiratory symptoms			
(Intercept)	1.6	0.10 to 3.10	.047
TIV^a^	0.21	−0.48 to 0.90	.554
Age	0.04	−0.09 to 0.16	.578
Number of systemic symptoms			
(Intercept)	−1.26	−3.27 to 0.74	.228
TIV^a^	1.13	0.21 to 2.06	.023
Age	0.17	−0.00 to 0.34	.062
Duration			
(Intercept)	0.96	−1.61 to 3.53	.47
TIV^a^	0.38	−0.80 to 1.57	.53
Age	0.14	−0.08 to 0.36	.22

a In comparison with hepatitis A vaccine.

### 3.2 Study 2: TLAIV vs. TIV

Over three influenza seasons between November 2012 and May 2015, participating individuals were monitored for influenza symptoms across 48 Hutterite colonies. Characteristics of the original study population have been outlined previously.^11^ Our primary analyses included children aged 36 months to ≤15 years of age who were randomized to receive TIV or TLAIV. Over three seasons, 166 PCR‐confirmed seasonal influenza infections occurred among 163 children who had been vaccinated with either TLAIV or TIV, across 36 Hutterite colonies.

The demographic characteristics of vaccinated (TLAIV or TIV) children with PCR‐confirmed influenza infections are shown in Table 4. The average age at time of infection was 9.42 years (minimum = 3.1 years, maximum = 15.0 years, SD 3.31 years), and 52.4% were female. Of 166 infections, 77 occurred in children who had received TIV, and 89 occurred in children who had received TLAIV. The number of infections that occurred per season was significantly different between the TIV and TLAIV groups (*P* = .029). The type of influenza strain causing the infection (H1N1, H3, or B) was also significantly associated with vaccine group (*P* < .001; Table 4).

**Table 4:** Demographic characteristics of PCR‐confirmed influenza infections among children from 2012 to 2015 across 36 Hutterite colonies

	All	Trivalent live attenuated Influenza Vaccine (TLAIV)	Trivalent Inactivated Influenza Vaccine (TIV)	*P*
	N = 166	N = 77	N = 89	
Age, mean (SD), y	9.42 (3.31)	9.34 (3.33)	9.50 (3.29)	.753
Male sex, N (%)	79 (47.6)	45 (50.6)	34 (44.2)	.504
Season (%)				
2012‐2013	70 (42.2)	45 (50.6)	25 (32.5)	.029
2013‐2014	63 (38.0)	26 (29.2)	37 (48.1)	
2014‐2015	33 (19.9)	18 (20.2)	15 (19.5)	
PCR‐confirmed Flu Strain (%)				
B	81 (48.7)	34 (38.2)	47 (61.1)	<.001
H3	45 (27.1)	32 (36.0)	13 (16.9)	
H1N1	37 (22.3)	21 (23.6)	16 (20.8)	
Unknown A	3 (1.8)	2 ( 2.2)	1 (1.3)	

On average, TLAIV recipients experienced a total of 3.13 symptoms (SD = 1.9 symptoms) for a mean duration of 5.1 days (SD = 4.2 days). In comparison with those in the TIV group, individuals who received TLAIV had significantly fewer total symptoms (mean difference = 1.2 total symptoms, *P* < .001, 95% CI: 0.55 ‐ 1.85), fewer respiratory (mean difference = 0.63 respiratory symptoms, *P* < .001, 95% CI: 0.271 ‐ 0.987), and fewer systemic symptoms (mean difference = 0.49 systemic symptoms, *P* = .01, 95% CI: 0.14‐1.01). Those vaccinated with TLAIV had a statistically equivalent duration as those who received TIV (*P* = .247, mean difference = 0.71 days, 95% CI −0.50‐1.93). Results are shown in Table 5. As seen in study 1, excluding repeat infections within children did not substantially change the results of our analysis; therefore, all infections were treated as independent and retained in the analysis.

**Table 5:** Symptom severity and duration, stratified by vaccine group, for PCR‐confirmed influenza infections among children from 2012 to 2015

	Intervention Group		95% CI	*P*
	Live attenuated Influenza Vaccine (LAIV)	Inactivated Influenza Vaccine (IIV)		
Number of symptoms, mean (SD)	3.13	4.34	0.554 to 1.85	<.001
Number of respiratory symptoms, mean (SD)	1.79	2.42	0.271 to 0.987	<.001
Number of systemic symptoms, mean (SD)	1.35	1.92	0.139 to 1.01	.01006
Duration, mean (SD)	5.08	5.79	‐0.499 to 1.93	.247

Multivariable models demonstrated that when controlling for age, children with an H1N1 infection who received TLAIV had significantly fewer symptoms (overall, respiratory, and systemic) and a shorter duration of symptoms compared to those who received TIV (*P* < .05 for all outcomes). All H1N1 infections occurred during season 2 (2013‐2014) of study 2; therefore, the effect of season was not assessed. For those with influenza B infection, we observed that individuals who received TLAIV had fewer total symptoms (*P* = .015) and fewer respiratory symptoms (*P* = .016) compared to those who received TIV, but the number of systemic symptoms and duration of illness was statistically equivalent between the groups (*P* > .05 for both outcomes). There was no statistically significant interaction effect of influenza season (ie, antigenic match) on the relationship between vaccine group and influenza severity for influenza B; therefore, we did not retain the interaction term in our model. We did not observe a significant effect of vaccine group on number of symptoms or duration of symptoms for those with an H3 influenza infection (*P* > .05 for all outcomes), and this relationship did not vary by influenza season; therefore, as above, we did not retain the interaction term in our model. Model output results are shown in Tables 6, 7, 8.

**Table 6:** Multivariable linear regression model output for all PCR‐confirmed influenza H1N1 infections among children (N = 37) from 2012 to 2015, adjusted for age

Predictors	Estimates	95% CI	*P*
Number of symptoms			
(Intercept)	3.87	1.37 to 6.38	.005
LAIV^a^	−2.2	−3.75 to −0.65	.009
Age	0.08	−0.14 to 0.31	.476
Number of respiratory symptoms			
(Intercept)	2.19	0.87 to 3.51	.003
LAIV^a^	−1.16	−1.97 to −0.34	.009
Age	0.05	−0.07 to 0.17	.408
Number of systemic symptoms			
(Intercept)	1.68	0.09 to 3.28	.046
LAIV^a^	−1.04	−2.03 to −0.06	.046
Age	0.03	−0.11 to 0.18	.664
Duration			
(Intercept)	7.21	3.01 to 11.40	.002
LAIV^a^	−3.36	−5.96 to −0.76	.016
Age	−0.12	−0.50 to 0.26	.549

a In comparison with TIV.

**Table 7:** Multivariable linear regression model output for all PCR‐confirmed influenza B infections among children (N = 81) from 012 to 2015, adjusted for age

Predictors	Estimates	95% CI	*P*
Number of symptoms			
(Intercept)	3.51	2.09 to 4.93	<.001
LAIV^a^	−1.1	−1.96 to −0.23	.015
Age	0.08	−0.06 to 0.22	.281
Number of respiratory symptoms			
(Intercept)	1.94	1.17 to 2.71	<.001
LAIV^a^	−0.59	−1.07 to −0.12	.016
Age	0.05	−0.03 to 0.12	.227
Number of systemic symptoms			
(Intercept)	1.57	0.59 to 2.55	.002
LAIV^a^	−0.5	−1.10 to 0.10	.104
Age	0.03	−0.07 to 0.13	.543
Duration			
(Intercept)	5.85	3.23 to 8.48	<.001
LAIV^a^	0.74	−0.86 to 2.34	.368
Age	−0.06	−0.32 to 0.20	.653

a In comparison with TIV.

**Table 8:** Multivariable linear regression model output for PCR‐confirmed influenza H3 infections among children (N = 45) from 2012 to 2015, adjusted for age

Predictors	Estimates	95% CI	*P*
Number of symptoms			
(Intercept)	3.07	1.12 to 5.01	.004
LAIV^a^	−0.89	−2.20 to 0.42	.191
Age	0.15	−0.02 to 0.32	.094
Number of respiratory symptoms			
(Intercept)	1.47	0.24 to 2.70	.024
LAIV^a^	−0.3	−1.13 to 0.53	.48
Age	0.08	−0.03 to 0.19	.146
Number of systemic symptoms			
(Intercept)	1.6	0.23 to 2.96	.027
LAIV^a^	−0.59	−1.51 to 0.33	.218
Age	0.07	−0.05 to 0.19	.276
Duration			
(Intercept)	6.56	2.47 to 10.65	.003
LAIV^a^	−1.89	−4.65 to 0.87	.186
Age	0.09	−0.27 to 0.45	.634

a In comparison with TIV.

## 4 DISCUSSION

Using prospective data from a randomized control trial, we present evidence that receiving the seasonal TIV does not attenuate the symptoms of disease in the context of vaccine failure among children in a community setting. Our analyses contribute two main findings. First, children with PCR‐confirmed influenza who received TIV did not experience fewer symptoms, or a shorter duration of symptoms, than children who received a control vaccine. This finding is supported by a previous study by Ng et al, which demonstrated that receipt of trivalent inactivated influenza vaccine did not reduce influenza symptoms or viral shedding in children who developed an influenza infection.^6^ However, unlike our study, the study by Ng et al only assessed vaccine‐matched influenza infections.^6^ In contrast, our study assessed both vaccine‐matched and unmatched influenza infections, and we found that when comparing hepatitis A to TIV, the antigenic match of the influenza vaccine and the circulating strain may in fact modify the relationship between influenza severity and vaccine group, specifically for influenza B infections. Second, when comparing recipients of TLAIV to TIV, children who were randomized to the TLAIV group had an attenuated course of disease when compared to those randomized to TIV, resulting in fewer symptoms overall. Furthermore, compared to children randomized to the TIV group, children who received TLAIV appeared to have significantly shorter symptom duration, and fewer symptoms for influenza H1N1, but not for influenza B or H3 infections. The relationship between severity of influenza infection among those who received TLAIV, compared to TIV, also appeared to be stable across seasons for all influenza strains.

The efficacy of the influenza vaccine is affected by not only host factors, but also dynamic epidemiological factors, such as antigenic match between the seasonal influenza vaccine and circulating strains each year.^8^ It therefore follows that receiving an influenza vaccination may not confer equal protection or attenuation of symptom severity across influenza strains. The strain‐specific differences observed in our analysis can in part be explained based on the antigenic match retrospectively observed between the vaccine and circulating streams for each season, as described in previous literature.^20^ For seasonal H1N1 and H3 viruses, the seasonal influenza vaccination was well matched in seasons 1 and 3.^21^, ^22^ For influenza B viruses, there was a lineage mismatch in season 1,^21^ but a match in season 3.^22^ The antigenic match data are consistent with our findings that, in comparison with those who received hepatitis A vaccine, seasonal influenza vaccination with TIV was associated with reduced symptom severity parameters among those with seasonal H1N1 and H3 viruses in seasons 1 and 3, and among those with influenza B in season 3 only. We also observed that children vaccinated with TIV who were infected with pandemic H1N1 in 2009 experienced a significantly greater number of total and systemic symptoms than those who were vaccinated with the control vaccine. This finding is supported by a study by Skowronski et. al, demonstrating that prior receipt of TIV was associated with a higher risk of pH1N1 illness and a higher risk of requiring medical attention, compared to those with pH1N1 who did not receive TIV.^23^ The biological mechanism of this risk is not completely understood.^24^ For study 2, LAIV and TIV formulations covered the same influenza strains each season; therefore, differences in symptom severity outcomes observed between vaccine groups may be attributable to factors other than antigenic match, such as vaccine type; however, recent studies comparing TLAIV and TIV vaccine efficacy have not supported this hypothesis.^11^

The effect of influenza vaccination on severity of infection remains controversial. Current literature suggests that the relationship between influenza vaccination and disease attenuation may vary between populations, clinical context, infection severity, and influenza strain. For instance, a 2014 study demonstrated that influenza vaccination reduced perceived symptom severity across eight systemic, upper respiratory, and lower respiratory symptoms only in adults > 65, but not in younger adults.^14^ Given that younger children were over‐represented in our study, we were unable to assess the association in adults aged 65 and over. Furthermore, a different study by Arriola et al^15^ showed that receipt of influenza vaccine conferred a protective effect on influenza severity among a cohort of hospitalized adults. This study measured rates of death, intensive care unit (ICU) admission, and hospital and ICU length of stay as a proxy for severity. In contrast, our study quantified severity using different measures in a community‐based out‐patient population, and furthermore, participants in our study were blinded to vaccine allocation; therefore, our results are not directly comparable.

A limitation of this analysis is that data were not available to quantify perceived severity, and therefore, we could not implement existing standardized scales to measure symptom severity. Various severity measures are used across influenza research, making direct comparison of results challenging. For instance, Hayden et al published a standardized, immunologically validated symptom severity scale for seasonal influenza.^19^ This self‐reported symptom severity scale uses a 4‐point scale (0 to 3) to score each influenza symptom, and symptoms are then collapsed into groups (systemic, upper respiratory, or lower respiratory symptom). Similarly, Van Wormer et al measured the self‐reported severity of eight symptoms (two URTI, two LRTI, and four systemic) on a scale from 0 to 3 and summed these scores for a maximum score of 24.^14^ Unlike Hayden et al,^19^ we did not collect data on hoarseness and chest discomfort; therefore, data on respiratory symptoms were not further stratified into URTI and LRTI in our study. Influenza vaccine efficacy studies among children that evaluate severity‐stratified end points use different outcome measures; for instance, studies by Ambrose et al and Jain et al define “moderate‐to‐severe” influenza as body temperature >39°C, physician‐confirmed otitis media, LRTI, or serious extra‐pulmonary manifestations. In both studies, all other laboratory‐confirmed influenza cases were categorized as “mild.”^25^, ^26^ In contrast to the aforementioned approaches, we did not measure self‐reported symptom severity. Instead, we used objective binary measures to quantify symptom presence/absence among participants, and defined severity as the summed number of symptoms, or symptom duration. It is possible that the presence of multiple symptoms may not be perceived as increased infection severity. Further analyses are required to determine whether our severity measure correlates with patients’ self‐reported perception of infection severity. A second limitation of this analysis is that the randomized population was limited to healthy children. Further investigation is required to prospectively validate the association between influenza vaccine type and subsequent infection severity among other patient populations, such as adults and high‐risk children.

One key strength of this analysis is the use of data from a blinded randomized control trial. These data include detailed demographic information, laboratory data, and daily symptom data collected prospectively for all study participants. By extracting data from a trial that required blinding and equivalent follow‐up in both intervention groups, our analysis was less vulnerable to bias, compared to studies that use a test‐negative approach to assess the severity of symptomatic influenza.^16^ In test‐negative studies, patients typically present to an emergency department with ILI. Patients then receive a laboratory influenza test to confirm diagnosis and are then questioned about their seasonal influenza vaccination status. Bias may arise in these studies due to unmeasured confounders associated with both exposure and outcome, or due to the effect of vaccination on healthcare‐seeking behavior.^17^ Our use of RCT data in our analysis minimized the risk of both selection bias and bias due to confounding factors, allowing us to objectively estimate influenza severity in the context of vaccine failure.

In conclusion, our study shows that in a community setting, healthy children vaccinated against influenza do not experience significant vaccine‐associated symptom attenuation, compared to those who received a control vaccine. When comparing influenza vaccine types, TLAIV does appear to provide superior vaccine‐associated attenuation of symptom severity than TIV for influenza A/H1N1 and influenza B. The relationship between vaccination and symptom severity in those who develop seasonal influenza appears to vary by demographics, clinical context, and flu strain. The widely held dictum that people who receive the influenza vaccine experience less severe symptoms does not appear to be generalizable to all patient populations and may vary from season to season based on vaccine match. Therefore, clinical setting and patient‐specific factors must be considered when evaluating the potential protective effect of the influenza vaccination in the context of vaccine failure.
